# Founder lineages in the Iberian Roma mitogenomes recapitulate the Roma diaspora and show the effects of demographic bottlenecks

**DOI:** 10.1038/s41598-022-23349-9

**Published:** 2022-11-04

**Authors:** Julen Aizpurua-Iraola, Aaron Giménez, Annabel Carballo-Mesa, Francesc Calafell, David Comas

**Affiliations:** 1 Departament de Medicina i Ciències de la Vida, Institut de Biologia Evolutiva (CSIC-UPF), Universitat Pompeu Fabra, 08003 Barcelona, Spain; 2 Facultat de Sociologia, Universitat Autònoma de Barcelona, Barcelona, Spain; 3 Facultat de Geografia i Història, Universitat de Barcelona, Barcelona, Spain

**Keywords:** Population genetics, Biological anthropology

## Abstract

The Roma are the largest ethnic minority in Europe. With a Northwestern Indian origin around ~ 1.5 kya, they travelled throughout West Asia until their arrival in Europe around the eleventh century CE. Their diaspora through Europe is characterized by population bottlenecks and founder events which have contributed to their present day genetic and cultural diversity. In our study, we focus on the effects of founder effects in the mitochondrial DNA (mtDNA) pool of Iberian Roma by producing and analyzing 144 novel whole mtDNA sequences of Iberian Roma. Over 60% of their mtDNA pool is composed by founder lineages of South Asian origin or acquired by gene flow during their diaspora in the Middle East or locally in Europe in Europe. The TMRCA of these lineages predates the historical record of the Roma arrival in Spain. The abundance of founder lineages is in contrast with ~ 0.7% of autochthonous founder lineages present in the non-Roma Iberian population. Within those founder lineages, we found a substantial amount of South Asian M5a1b1a1 haplotypes and high frequencies of West Eurasian founder lineages (U3b1c, J2b1c, J1c1b, J1b3a, H88, among others), which we characterized phylogenetically and put in phylogeographical context. Besides, we found no evidence of genetic substructure of Roma within the Iberian Peninsula. These results show the magnitude of founder effects in the Iberian Roma and further explain the Roma history and genetic diversity from a matrilineal point of view.

## Introduction

The Roma, also known by the misnomer of ‘Gypsies’, are the largest ethnic minority in Europe, with an estimated population size of 10 to 12 million people^1^. The term Roma encompasses a mosaic of diverse groups (Roma sensu* strictu*, Sinti, Kale, Romanichels, and others) that differ genetically, culturally, linguistically and historically. However, genetic and linguistic studies have placed the origin of all Roma in the Northwestern part of the Indian subcontinent^2–4^. Overall, the Roma diaspora has not been historically well documented. Nevertheless, historical evidence seems to suggest they travelled through Persia and arrived to Armenia around the ninth century CE. Strong linguistic influence indicates that their sojourn in Armenia may have been particularly extended^5^. Historical records show that the Seljuk invasions in Armenia that caused the well-known displacement of the Armenians, could also have forced the Roma ancestors to follow the Anatolian routes towards Constantinople and the Balkans, where the presence of Roma was recorded in the mid-eleventh century^6,7^. In Europe, Roma settled in the Southern Balkans for around 200 years and stayed in the region under the Ottoman empire. The expansion of the latter towards central Europe, however, triggered the spread of Roma nomadic groups throughout different European territories^6^. In addition to the nomadic tradition of Roma, the persecution and social exclusion suffered in many territories played a key role in their dispersion and formation of the different Roma ethnolinguistic groups in Europe. Some Roma moved to the Danubian Principalities (nowadays Romania, Moldavia and Hungary) where they were forced to slavery and became the Vlax Roma, while some other groups, such as the Romungro Roma, moved to the Austro-Hungarian empire. Finally, many other small groups took northern or western routes and spread throughout Northern, Central and Western Europe^7,8^. Finally, the Roma arrived in the Iberian Peninsula in the fifteenth century, according to historical records mentioning the presence of Roma in 1425 in Zaragoza^9^. In the Iberian Peninsula in 1469, the kingdoms of Castille and Aragon unified and started to seek cultural homogeneity within their borders^10^. The Iberian Roma, together with the Muslim and Jewish communities, conflicted with the desired cultural and religious (Catholic) homogeneity^10,11^. Therefore, a series of laws were enacted to force Muslims and Jews to convert or be deported, and nomadic Roma to settle^11,12^.

The understanding of the present complexity of Roma and their history has been undertaken from different disciplines. Molecular anthropological studies first proposed a South Asian genetic component in Roma due to the presence of shared genetic diseases with Indian and Pakistani patients^13,14^. Afterwards, thanks to genomewide data, their origin was placed 1500 years ago in the Punjab^3,4^. In the demographic models best fitting the genomic evidence, the proto-Roma had a reduced population size, and were genetically nearest to the present-day Punjabi groups with the least West-Eurasian ancestry^4,15,16^. The Indian origin of Roma is also reflected in their uniparentally transmitted genomic pools, since they harbor South Asian mitochondrial DNA (mtDNA) M (M5, M35, M18 and M25) and Y-chromosome H1a1a4b2 lineages^3,17,18^.

After the out of India event, which implied a very strong founder effect in their genomes, Roma admixed extensively with non-Roma populations, and underwent multiple bottlenecks along their diaspora, leading to the formation of the present-day Roma^4,15,16,19^. A moderate influence of a Middle Eastern component and a slightly higher genetic impact from Caucasian populations have been observed in genome wide genetic studies^15,20,21^. This agrees with a rapid dispersal of Roma through Persia and the impact of the Caucasus region proposed by historical and linguistic studies. Besides, a Balkan genetic footprint has also been observed in all Roma populations^15,19^. This component forms a gradient that is correlated with the distance to the Balkans, being the Bulgarian, Greek and Serbian Roma those with highest Balkan proportions (45%) in contrast with the Lithuanian, Estonian or Iberian Roma, who carry the Balkan component at ~ 25%^19^. This also agrees with historical records, which together point to a Roma dispersal in Europe from the Balkans. In addition to the Balkan component, Roma populations also show different patterns of admixture with their respective host populations, which, added to the population bottlenecks (and founder events) and/or social endogamic practices, make up for the observed genetic heterogeneity of European Roma^19^. The Roma demographic history has also shaped their functional genomic variation, since founder effects increased high-frequency deleterious variants^22^.

Iberian Roma are the largest Roma population outside the Balkan/Eastern Europe and represent the westernmost Roma expansion in Europe^1^. Yet, there are still few studies focusing on the Iberian Roma. A recent genome-wide study carried out on different Roma populations suggested geographic genetic substructure within the Iberian Peninsula with differing levels of ancestry proportions and inbreeding, understood as mating of individuals closely related through ancestry^19^. Regarding studies about uniparental markers, the multiple founder events left also an imprint in the Roma maternal lineage frequencies. Martinez-Cruz et al. 2016 identified the main Roma specific mtDNA founder lineages by sequencing the mitochondrial control region. Later, whole mitogenomes were used to increase the phylogenetical detail of a few of these lineages present in Iberian Roma (such as M5a1b1a1, U3b1c or H88a)^23^.

Uniparental markers provide a unique picture of sex-specific patterns of human migration and admixture. However, the Iberian Roma mtDNA pool has not been fully inspected. Although the presence of founder lineages in Iberian Roma is known to be significant^24^, many features still remain unknown, particularly when and where these lineages were acquired by Roma. These questions were investigated in previous studies, but were limited by small sample sizes and the suboptimal resolution provided by the mtDNA control region^23–25^. Besides, it remains to be explored whether founder lineages are restricted to populations that have undergone bottlenecks, or if, on the contrary, they also appear in general European populations.

Therefore, in the present study we analyze the mtDNA pool of Iberian Roma, focusing on the Roma founder lineages present in the proto-Roma population or acquired by admixture throughout their history and subsequently surging by drift. We also explore the mitochondrial genetic substructure of Roma within the Iberian Peninsula, in addition to the possible functional implications that demographic processes could have had in the Roma mitochondrial founder lineages.

## Methods

### Samples and sequencing

We sequenced the whole mitogenome of 144 Iberian Roma volunteers extracted from saliva samples. The collection of the saliva samples from the volunteers was performed under the umbrella of the “*El Camí del Poble Gitano: una història de Diversitat*” project^26^ in collaboration with the Roma FAGiC association (*Federació d’Associacions Gitanes de Catalunya*). All participants self-identify as Iberian Roma and appropriate written consent was obtained from all donors (Supplementary Information). This study has been approved by our local IRB (Comitè d’Ètica de la Investigació, Parc de Salut Mar, references 2016/6723/I, on June 7th 2016; and 2019/8900/I, on Jan. 15th, 2020), and preliminary results were presented to the Roma community in a meeting organized by the FAGiC on February 1st 2019 in Barcelona. All methods in this study were performed following the standard guidelines and regulations.

PCR amplifications were performed in four different fragments under the same conditions (Tables S1 and S2). Nextera XT libraries were prepared and sequenced following the Illumina mtDNA Genome Guidelines^27^ and the Illumina MiSeq Guidelines^28^.

### Sequence processing

Sequences were processed according to the GATK best practices steps^29^. First, an initial quality assessment was carried out with FastQC^30^ and then BWA 0.7.15^31^ was used to map the raw reads to the revised Cambridge Reference Sequence (rCRS)^32^. The PCR duplicates were removed with Picard tools^33^, base quality scores were recalibrated, and a final quality report was obtained by Qualimap^34^. Finally, haplotypes were called using GATK haplotype caller^35^.

### Statistical analyses

Haplogroups were determined with haplogrep v.2^36^ using phylotree v.17 plus the additional haplogroups defined by Dür et al.^37^. Molecular diversity and summary statistic indexes were calculated with the pegas^38^ package in R and poppr^39^ was used to compute AMOVA and *F*_*ST*_ distances by geographical region. Coalescent ages were obtained by calculating ⍴ and σ^40^, assuming a mutation rate of 2.355 × 10^−8^ substitutions per nucleotide per year, taking into account purifying selection as in^41^.

### Phylogeographical analysis

We define founder lineages in Iberian Roma as groups of more than four mitochondrial sequences of the same haplogroup (as defined in phylotree.org build 17^42^), that share one or more mutations (not accounting for the hypervariable positions listed in Soares et al. 2009), and that are restricted exclusively to Roma individuals. To verify whether these founder lineages are exclusive to the Iberian Roma, Blast was used to search Genbank for sequences sharing the highest identity percentage.

In order to compare the relevance of these founder lineages within the Iberian Roma, we repeated the analysis in a dataset of over 1,000 non-Roma Spanish sequences^43,44^. First, the whole non-Roma Spanish reference dataset was analyzed looking for haplogroups reaching a relative frequency of 0.0278, which is the value corresponding to the threshold set for the Roma (4/144, where 144 is the Roma sample size). Then, we subdivided the non-Roma sample into geographical groups of a size closer to that of the Roma sample (Table S3), inspected them for founder lineages with the same relative frequency threshold but n > 2, and run BLAST in Genbank to retrieve the most similar sequences. With all the downloaded sequences added to the Spanish ones, we constructed the Median Joining Phylogenetic tree with the Network 10 software and built the tree-like representations manually.

### Pathogenicity measure

We used the pathogenicity scores predicted by the MutPred software for all possible amino acid change in the mtDNA coding region available in the Table S3 in Pereira et al.^45^. We then compared the distribution of the pathogenicity scores between the founder lineages and non-founder lineages by using a Mann–Whitney U test.

## Results

### Overall mitochondrial diversity within the Iberian Roma

We sequenced the mitogenome of 144 individuals from the Iberian Roma population at a mean coverage per individual of 624X (Fig. S1). The Iberian Roma show 72 different mitochondrial haplotypes and lower values of diversity in comparison to any of the geographical regions of the non-Roma Spanish population (Table S4).

**Figure 1:**
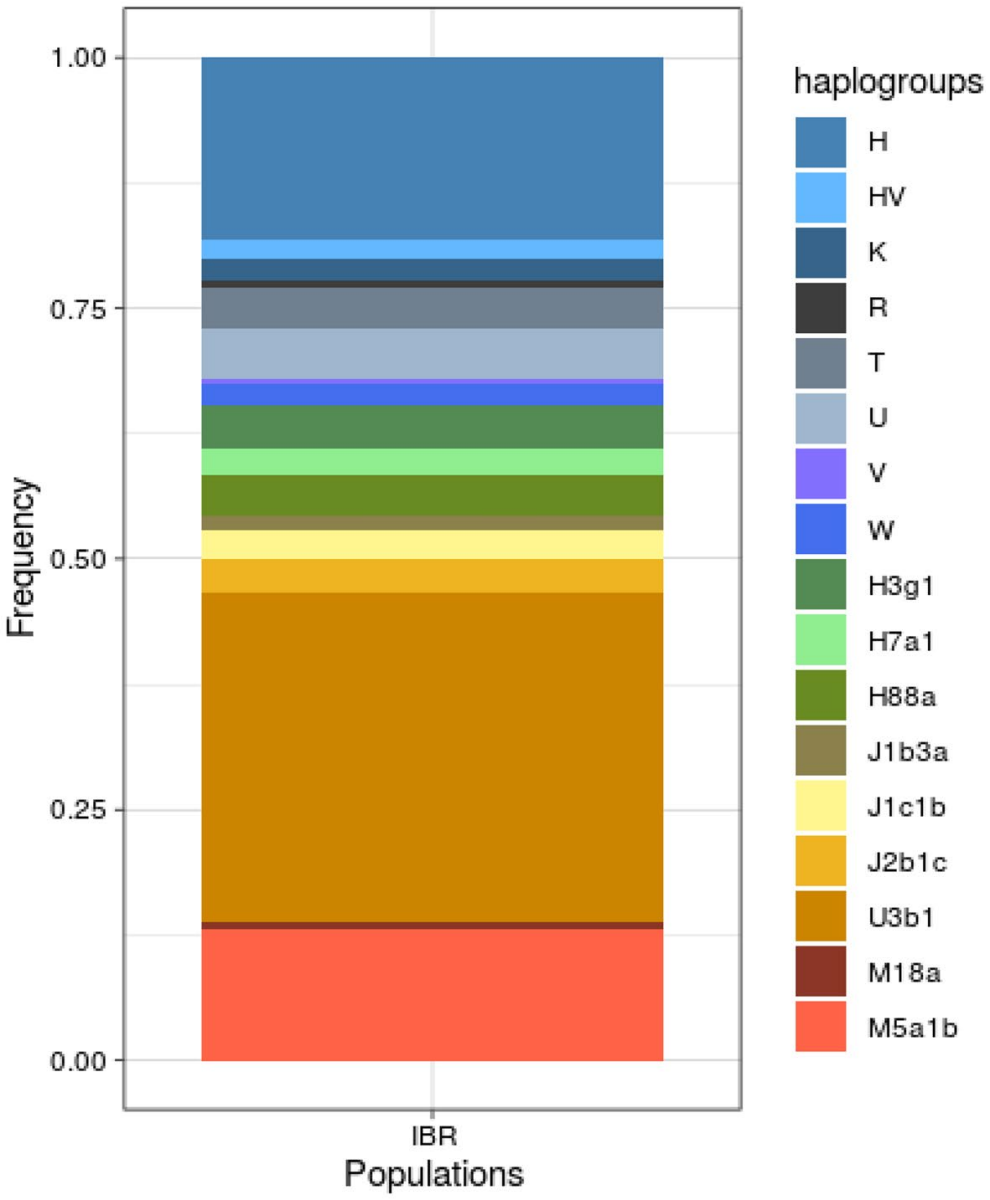
Haplogroup composition in Iberian Roma. Founder lineages are detailed to the sub-haplogroup level.

Our samples show 20 (13.9%) M South Asian haplotypes, mainly represented by M5a1b haplogroup sequences, and 124 (86.1%) West Eurasian haplotypes. Besides, our samples show 51% of West Eurasian founder lineages that had previously been described either by analysis of mitochondrial control regions (U3, H7, J1b3 or J1c1) or whole mitogenome analysis (H88a, U3b1c)^23,24^. In addition to these previously observed Roma founder lineages, we were also able to detect the presence of some new founder lineages within haplogroups H3g1 and J2b1c (Fig. 1). Overall, two founder lineages of South Asian origin were carried by 13.9% of the individuals of our Iberian Roma sample, while seven West Eurasian founder lineages reached a joint 51.4% population frequency.

### Roma founder lineages

**Figure 2:**
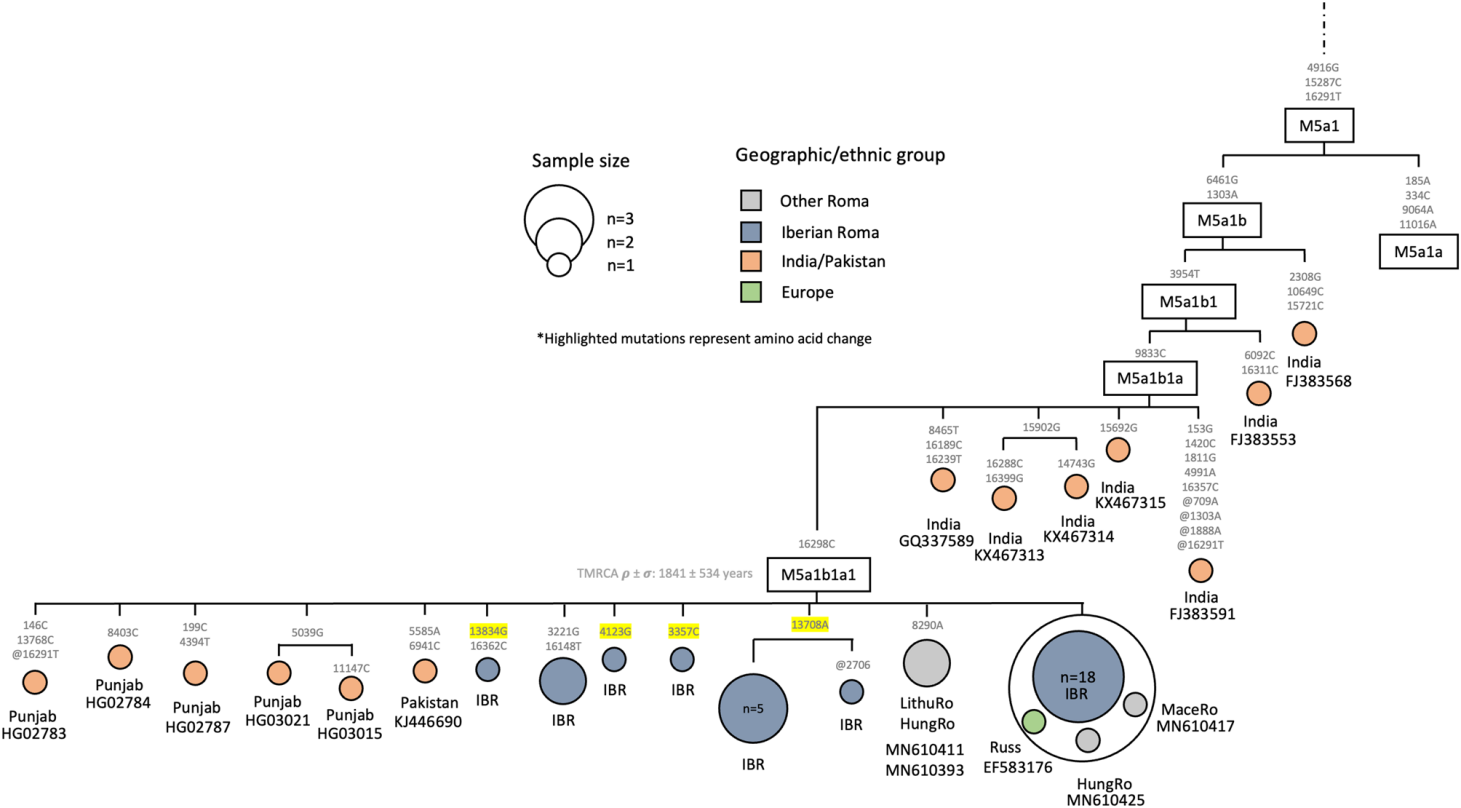
Maximum parsimony tree of the haplogroup M5a1b1a1 Roma mitogenomes. The mtDNA variants are indicated along the branches of the phylogenetic tree. The ‘@’ symbol represents a back mutation, and the highlighted mutations represent non-synonymous mutations. The IBR tag refers to Iberian Roma, and the ‘LithuRo’, ‘HungRo’, and ‘MaceRo’ refer to Lithuanian, Hungarian and Macedonian Roma respectively.

Given their nearest neighbors, the M5a1b sequences within the Iberian Roma (13.2%) (Fig. 2) are clearly of South Asian origin; all Roma samples contain the mutations defining M5a1b1a1. However, within M5a1b1a1, some divergence is observed between Roma samples, evidencing previously unobserved diversity levels within the maternal genetic pool of the proto-Roma population. In addition, we observe five Punjabi and one Pakistani individuals clustering with all the Roma samples within the M5a1b1a1 branch. This is further evidence that this lineage and the proto-Roma population have their origin somewhere around north-western India and Pakistan. The time to the most recent common ancestor (TMRCA) is 1.8 kya (σ = 0.53 kya). As for the remaining M South Asian lineages detected in a previous study^23^, M35 was absent in our samples, and we found only one M18 haplotype.

**Figure 3:**
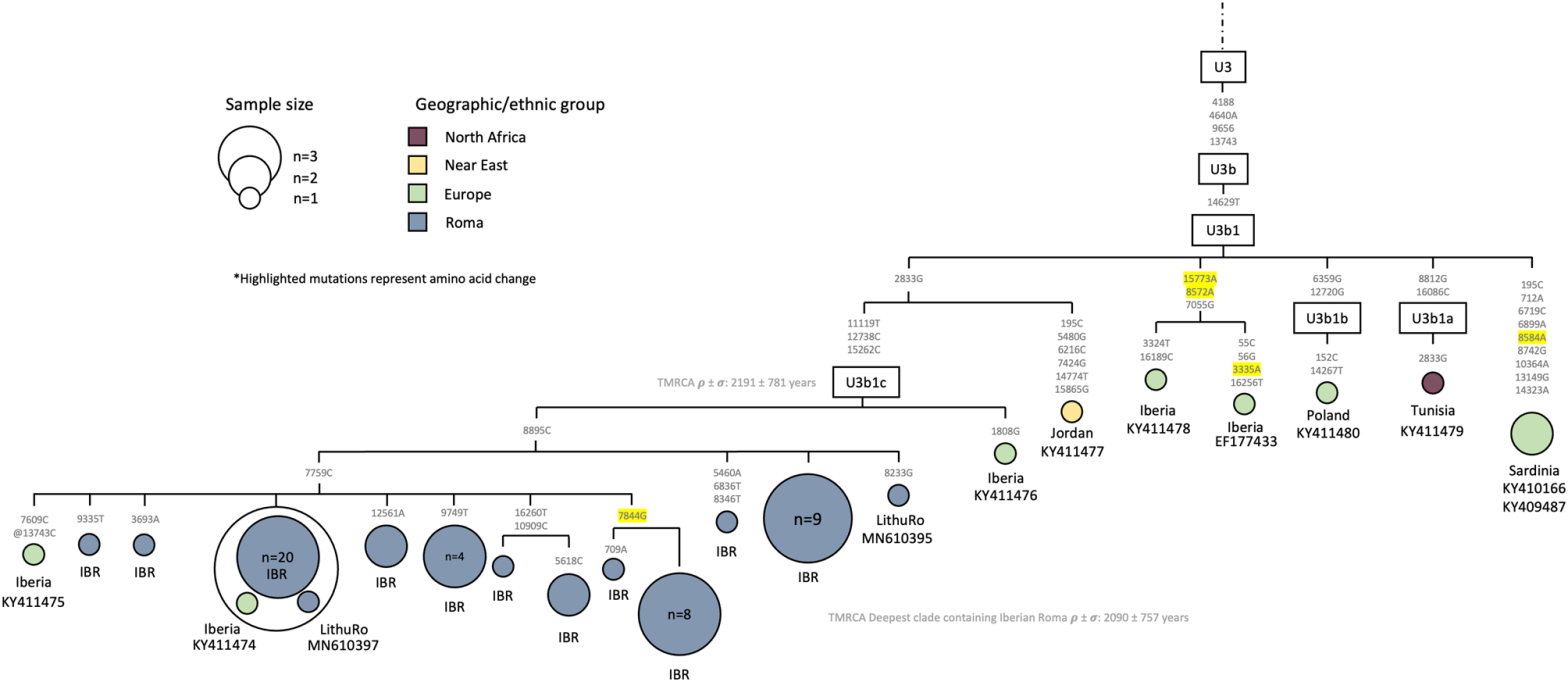
Maximum parsimony tree of the haplogroup U3b1c Roma mitogenomes. The mtDNA variants are indicated along the branches of the phylogenetic tree. The ‘@’ symbol represents a back mutation, and the highlighted mutations represent non-synonymous mutations. The IBR tag refers to Iberian Roma, and the ‘LithuRo’ refers to Lithuanian Roma.

The most frequent West-Eurasian founder lineage in the Iberian Roma is U3b1. In a previous study^23^, the phylogeny of the Roma U3b1 was further refined for five Iberian Roma individuals falling within the same U3b1 sub-branch (thereafter named U3b1c) and sharing the variants A2833G-T7759C-T8895C-C11119T-T12783C-T15262C. In our study (Fig. 3), 47 sequences (32.6%) belong to the U3b1 haplogroup. However, not all of them share the U3b1c defining variants, some of them lacking T7759C. The presence of an Iberian non-Roma individual with four out of six U3b1c defining mutations suggests that there might be even more unsampled diversity within this branch and that the definition of the U3b1c should be carefully revised. In addition to this sample, we observe another Iberian individual falling within all the U3b1c Roma samples which could be a signal of the contribution of Roma to the general Iberian population. The presence of U3b1c in other European Roma, and the fact that the closest lineage sharing the A2833G mutation was sampled in Jordan suggest that this lineage could have been acquired by Roma in the Middle East during their diaspora earlier than expected (TMRCA = 2.1 kya σ = 0.78 kya).

Each one of the rest of the founder lineages have a smaller presence in the mitochondrial pool, but together they represent 18.7% of the total lineages. Within the founder lineages previously described from the control region sequence^24^, we were able to refine the phylogeny of H7a1, J1b3 and J1c1. The H7a1 haplogroup (Fig. S2) is defined by C16261T, and its distribution is restricted to Central-Northern Europe. Four of our H7a1 Iberian Roma samples fall outside every sub-haplogroup nested within H7a1 and form a separate branch defined by the 14905A mutation. Additionally, one of them contains the 16148 T mutation.

**Figure 4:**
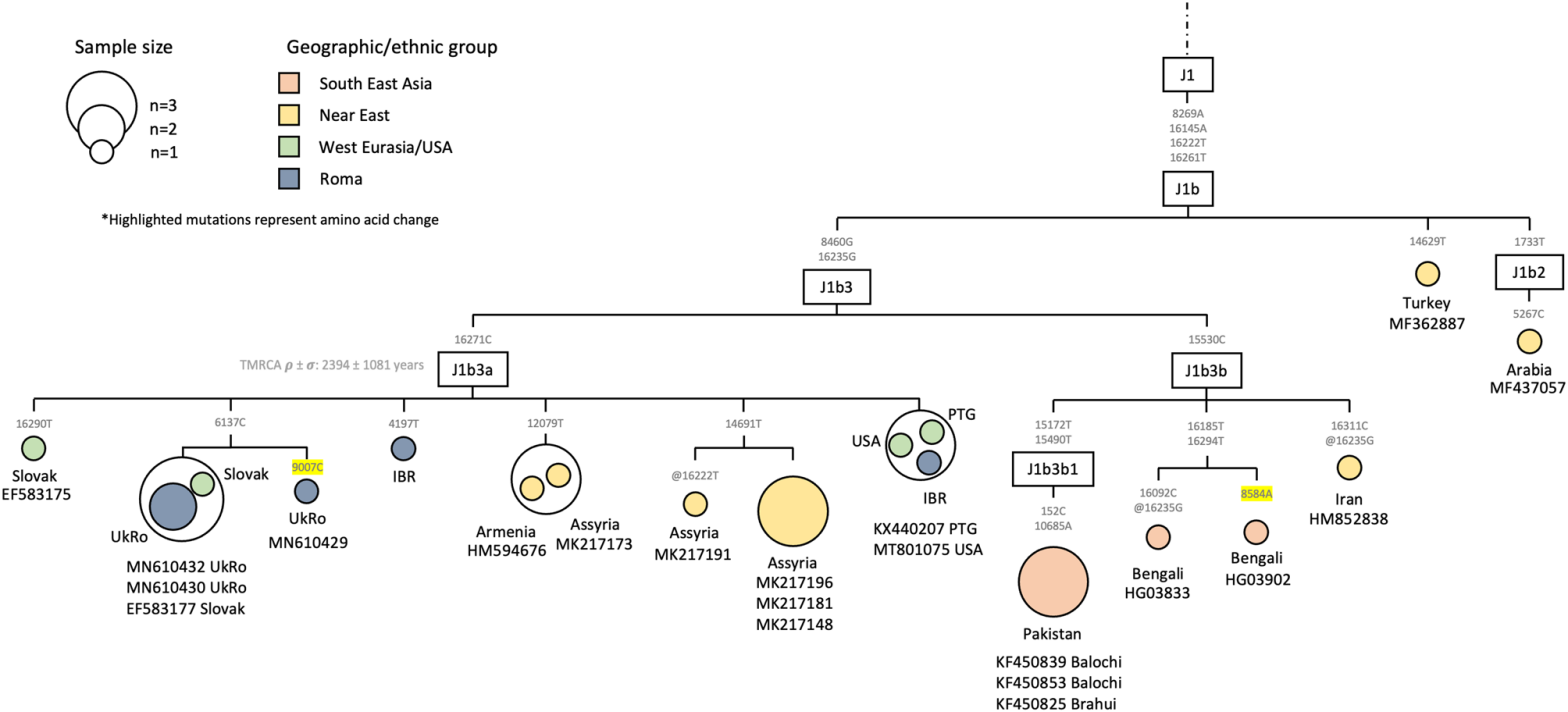
Maximum parsimony tree of the haplogroup J1b3a Roma mitogenomes. The mtDNA variants are indicated along the branches of the phylogenetic tree. The ‘@’ symbol represents a back mutation, and the highlighted mutations represent non-synonymous mutations. The IBR tag refers to Iberian Roma, the ‘UkRo’ refers to Ukrainian Roma and the 'PTG' refers to Portugal.

Haplogroup J1b3 (Fig. 4) is split into two sub-haplogroups, J1b3a and J1b3b. Roma samples all fall within the J1b3a branch and show four different haplotypes. Three Ukrainian Roma together with a Slovak individual share the 6137C mutation. The Iberian Roma individuals show two distinct haplotypes, one at the J1b3a root (together with one Portuguese and one US sample), and the other separated from it by the 4197T mutation. Besides, some branches of the J1b3a haplogroup are present in Armenian and Assyrian individuals.

J1c1b (Fig. S3) also harbors a Roma-specific founder lineage. Four of our Iberian Roma samples, together with one Macedonian Roma and one Hungarian Roma form a branch defined by 508G-9554A-14470C. All four Iberian Roma and the Macedonian Roma show the same haplotype while the Hungarian Roma additionally carries 10463C. The coalescent age for these sequences is 0.4 kya (σ = 0.4 kya).

H88a, described in a previous study^23^, was also found in six individuals within our Roma population and yields a coalescent age of 0.9 kya (σ = 0.5 kya) (Fig. S4).

Besides these haplogroups that had been previously described in the Roma, we discovered additional founder lineages, such as H3g1 (Fig. S5), which is present in five Iberian Roma individuals and is defined by 7419A-11563 T. Four of these sequences also contain the 3666A mutation, while another individual carries the 16153A mutation. The coalescent age of these sequences is 2.5 kya (σ = 2.1 kya).

**Figure 5:**
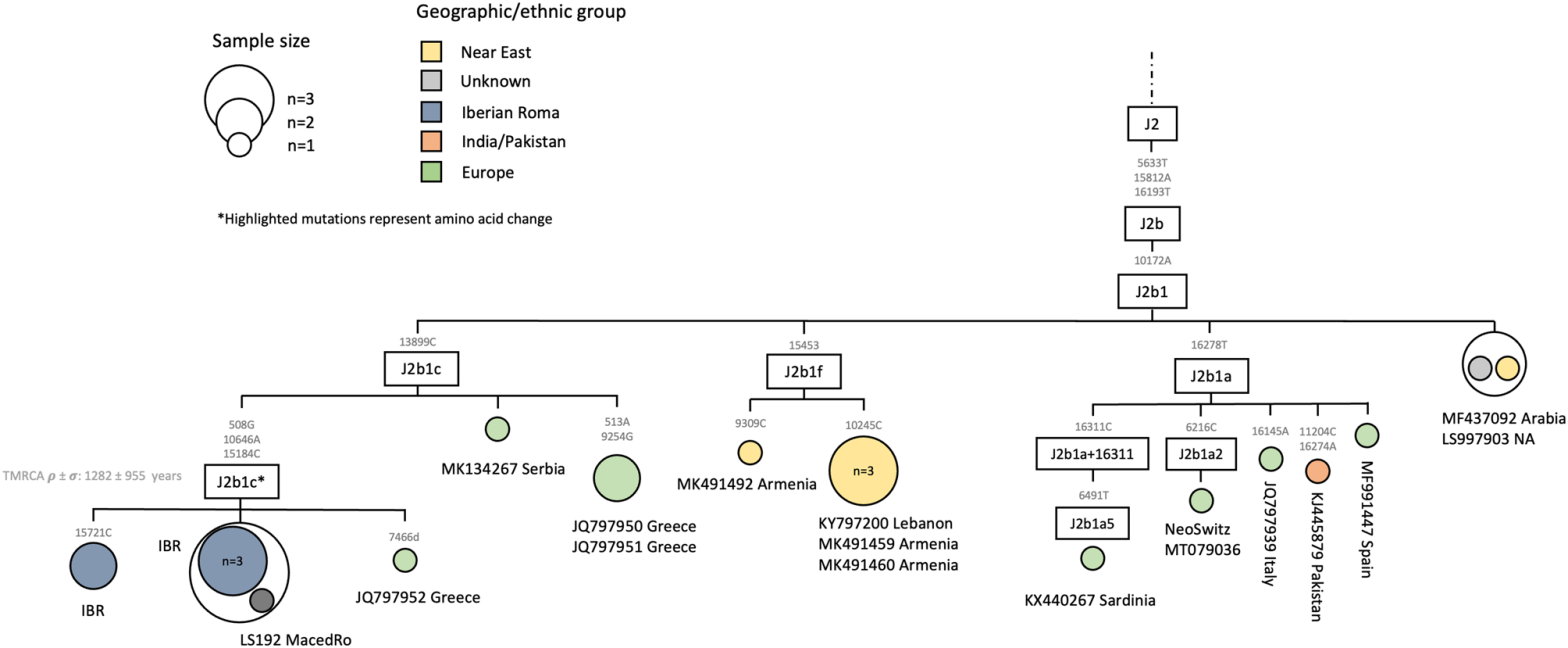
Maximum parsimony tree of the haplogroup J2b1c* Roma mitogenomes. The mtDNA variants are indicated along the branches of the phylogenetic tree. The highlighted mutations represent non-synonymous mutations. The IBR tag refers to Iberian Roma.

Finally, there are five sequences belonging to haplogroup J2b1c* (sensu stricto Dür et al. 2021) within our samples (Fig. 5). Together with a Greek and a North Macedonian Roma sample, they form a separate cluster defined by mutations 508G-10646A-15184C. The coalescent age of the branch is 1.2 kya (σ = 0.9 kya).

**Figure 6:**
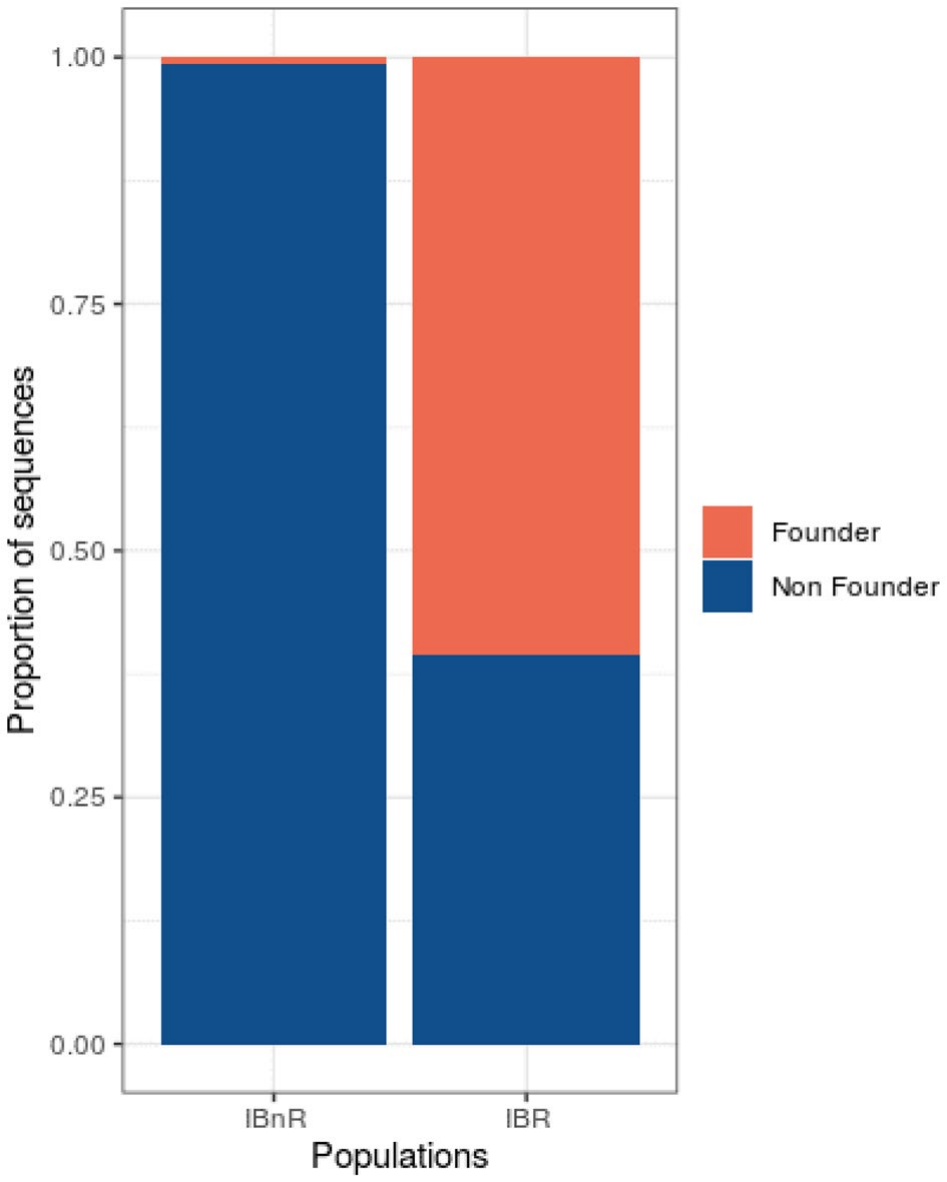
Proportion of population specific founder and non-founder lineages in the Iberian Roma (IBR) and Iberian non-Roma (IBnR) populations.

In order to determine whether the amount and overall frequency of founder lineages we found in the Iberian Roma was a feature presumably derived from their demographic history or was instead similar to what can be found in other populations, we looked for founder lineages in the dataset of the Spanish general population (see “Methods”, Figs. S6−S13). We observe that 8 out of 1,066 (0.7%) Spanish non-Roma sequences could be assigned to Spanish-specific founder lineages, while for the Iberian Roma dataset, 94 out of 144 (65.3%) sequences belonged to Roma founder lineages (Fig. 6). Within the Spanish non-Roma dataset, we identified some individuals (1.4%) belonging to a H1j1, a Basque characteristic lineage^46^ that were not considered due to the known particular demographic history of this population^47^ (Fig. S7). In Iberians other than Basques, we identified two possible founder lineages: one formed by three K1a4 sequences from Catalonia, and one formed by five T2 individuals from Andalusia and Catalonia (Figs. S11 and S13).

### Genetic substructure within Iberian Roma

The genetic structure of Iberian Roma within the Iberian Peninsula was analyzed by separating the samples according to the geographic origin (Fig. S14). Genetic diversity indexes were calculated for each of the regions (Table S3). The general haplogroup composition of the regional groups (namely, its classification into South Asian founder lineages, European founder lineages, and European non-founder lineages) was not significantly heterogeneous (p = 0.255, Pearson’s chi-squared test) (Fig. S15).

In addition, regional substructure in sequence composition was analyzed by means of AMOVA. The fraction of variation among the Iberian Roma sub-populations was − 0.41%, while for the Iberian non-Roma was 0.04%, both not significantly different from zero, which suggests homogeneity between the geographical regions both for the Iberian Roma and for the general population.

The amount of genetic diversity explained by the differences between the Roma and the non-Roma Iberian populations was 10.7% (p = 0.0001), as measured with AMOVA. This is a large value that could be explained by the dominance of the founder lineages in the Roma. Indeed, if all founder lineages were removed, that variance fraction dropped to 0.3% (p = 0.2). This implies that mtDNA sequences in Roma other than founder lineages are indistinguishable from non-Roma haplotypes and could be mostly the result of recent admixture with non-Roma Iberian.

### Mitochondrial deleterious mutational load

The deleterious mutational load in Iberian Roma was analyzed looking into the MutPred score of non-synonymous mutations in the coding region. We compared the predicted pathogenicity of the amino acid changes of Roma founder lineages with Roma non-founder lineages. The results show that, contrary to what could be expected, the Roma non-founder lineages show a higher mean pathogenicity comparing to the Roma founder lineages (Fig. S16). Although a higher presence of deleterious mutations could have been possible due to genetic drift during their diaspora, the mtDNA evidence shows that European lineages have a higher predicted pathogenicity than Roma founder lineages (p = 4.956 × 10^–5^, Mann–Whitney U-test), either compared to South Asian (p = 0.02) or European (p = 10^–4^) lineages (Fig. S17).

## Discussion

The genetic diversity and population history of the Roma have been assessed with both uniparental and genome wide data^3,23,24,48,49^. Mitochondrial DNA studies, however, have been mainly focused on control region sequences, and, in studies using whole mitogenomes (especially those focusing on North-Western Roma), sample size has been a limitation^23^. In the present study, we overcome this constraint by sequencing 144 new samples from Roma living in Spain.

Our results show lower mitochondrial diversity levels in Roma when compared to the Iberian general population, which agrees with previous studies and reflects the population bottlenecks undergone by the Roma population^24^. However, looking more in depth into the mtDNA pool, we observe that, besides the South Asian M lineages found in the Roma (13.9%), the remaining mtDNA haplotypes are evidence of the intense Roma admixture with West Eurasian populations along their out of India diaspora.

Regarding the South Asian Roma lineages, the reduced population size of the proto-Roma who underwent the out of India event is reflected in the low diversity levels observed. This is evident for the mitochondrial pool, where European Roma show a few different M sub-haplogroups (such as M5, M35 or M18), and it is even more noticeable in the Y chromosome diversity, where just the H1a1a4b2 and R1a-M780 lineages were carried out of India^17,50^. Most of our samples belong to the M5a1b1a1 haplogroup, which is shared with six South Asian samples from the Punjab region in north-western India and Pakistan. This suggests that the geographic origin of this lineage and the proto-Roma population was probably around the north-western part of the Indian subcontinent (assuming the present-day haplotype distribution in the region reflects the one before the departure of the proto-Roma), in agreement with previous studies^4,15^. The molecular dating for the origin of this lineage (~ 1800 ybp) predates the out of India event of the proto-Roma population, which has been estimated to be around 1500 ybp^15^.

The most abundant lineage in Iberian Roma is U3b1c, which is also present in Lithuanian Roma and, to a lesser extent, also possibly in Bulgarian and Greek Roma, considering the U3 lineages observed in control regions^24^. In contrast with previously published studies, Iberian Roma show higher diversity levels within this haplogroup. The presence of an Iberian non-Roma individual with four out of six U3b1c defining mutations suggests that there might be even more unsampled diversity within this branch, and that the definition of U3b1c should be carefully revised. The origin of this lineage is difficult to trace phylogeographically, since the few samples available related to the U3b1c lineage have an unspecific geographic distribution (Fig. 3). Still, the closest haplotype to U3b1c, sharing one mutation with it, was sampled in Jordan. Coupled with the relatively wide distribution among Roma and estimated TMRCA of 2.1 kya, which predates the Roma population history and is possibly due to the divergence of the adopted lineages, the most likely origin of U3b1c is in the Middle East. If that was indeed the case, over 30% of the Iberian Roma mtDNA pool would consist of a Middle Eastern lineage. This is in contrast with the modest Middle Eastern/Caucasian impact observed in studies using autosomal data, which is specially low in the Roma X chromosomes^19^. Further studies on mtDNA could shed light on the origin of U3b1c haplogroup, which could have reached a high frequency in Iberian Roma by drift events.

Additionally, J1b3a could also have a Middle Eastern origin, since it has also been found in Armenians and Assyrians (Fig. 4). This lineage, which we find for the first time in Western Roma, is moderately frequent in Roma from Bulgaria and Hungary and quite prevalent in Ukrainian Romungro Roma^24^.

Besides, we find J2b1c* lineages just to be present in non-Roma individuals from the Balkans besides the five Iberian Roma samples and a Macedonian Roma (Fig. 5). This might indicate that the lineage was obtained by Roma during their stay in the Balkan region, which agrees with the Balkan ancestry found in all European Roma^19^.

The rest of the founder lineages in Iberian Roma, comprising 13.9% of the sample, have a clear European origin, albeit a precise origin within the continent cannot be pinpointed. Still, given the patterns of haplogroup sharing with other Roma groups and the estimated lineage ages, it is probable that most of them were acquired soon after the Roma arrived in Europe rather than in the Iberian Peninsula itself.

Noteworthily, we have not found any North African autochthonous haplotypes such as M1 or U6 within our sample. This agrees with previous results that imply that the Roma entering the Iberian Peninsula through North Africa was rather unlikely^3,4^.

After having quantified the differences in the frequency of founder lineages between Iberian Roma and the general Spanish population, the results indicate that the presence of mitochondrial founder lineages might not be a common feature in general European populations but rather a characteristic feature of populations that have undergone certain demographic processes. Founder effects caused by population bottlenecks throughout the Roma diaspora in Europe have been the main reason for the high frequency of founder lineages in the Roma mitochondrial pool. Besides, the maintenance of a genetic continuity in Roma, caused by sociocultural factors (social exclusion, endogamous marriages), might have also played a role in the patterns observed in the Iberian Roma mitochondrial pool, as well as in the negligible presence of Roma founder lineages observed in the general Spanish population.

The evaluation of the genetic substructure within the main Iberian geographic regions reveals that the Iberian Roma are a genetically homogeneous population from the maternal point of view. We could not detect regional differences in the proportion of South Asian founder lineages, European founder lineages, and European non-founder lineages; and AMOVA did not detect any significant variation among the different regions either. The traditional itinerant lifestyle of Roma was partially also impelled by the social exclusion they suffered historically. The highest expression of Spanish Roma repression is the Great Roma Round-Up in 1749. The Great Round-up was a raid organized by the Spanish monarchy with the objective of imprisoning and/or forcing Iberian Romani into labor. This event and overall, the repressive legislation against Roma had a strong impact in the loss of the Romani language and culture, propelled the anti-gypsyism in Spain and caused the displacement of many Roma families^51^. This may have contributed to the lack of genetic substructure in the current Iberian Roma population.

The use of complete mitochondrial sequences enables the analysis of the mitochondrial coding regions, and therefore, also, the analysis of functional implications that demographic history can have in populations. Population bottlenecks and subsequent founder effects have increased the deleterious mutational load in the protein coding part of the genome^22^. Thus, we tried to explore the deleterious mutational load of Roma mitochondrial lineages by comparing the predicted pathogenicity of the non-synonymous mutations for South Asian founder lineages, West-Eurasian founder lineages and European non-founder lineages. Interestingly, South Asian lineages and West-Eurasian founder lineages have a lower mean predicted pathogenicity compared to the non-founder lineages, contrasting with what it was observed in the autosomal exomes. This might be due to the different selection regime affecting mtDNA, in which often heteroplasmic mutations can cause disease and are selected against, while most mutations in autosomal genes are recessive.

The present study shows that founder effects along the Roma diaspora have strongly influenced their mtDNA pool. Furthermore, the phylogeographical analysis of the founder lineages enables the recapitulation of their diaspora, starting from Northwestern India all the way to Europe. The Roma diaspora is an underdocumented event and their relationship with other populations throughout their diaspora is something future genome-wide studies could focus on to help in filling up the gaps in the knowledge about the Roma history.

## Supplementary Information

Supplementary Information.

## Data Availability

The mitochondrial genomes produced in this study are available on GenBank with the accession numbers: ON155447—ON155590.
