# Supplementary material for: Founder lineages in the Iberian Roma mitogenomes recapitulate the Roma diaspora and show the effects of demographic bottlenecks
Source: Sci Rep. 2022 Nov 4;12:18720. doi: 10.1038/s41598-022-23349-9 (PMC9636147; doi:10.1038/s41598-022-23349-9)
Supplement: Supplementary file 1 — Supplementary Information. [file 41598_2022_23349_MOESM1_ESM.pdf]

SUPPLEMENTARY MATERIAL

Founder lineages in the Iberian Roma mitogenomes recapitulate the Roma diaspora and show the effects of demographic bottlenecks

Julen Aizpurua-Iraola[1], Aaron Giménez[2], Annabel Carballo-Mesa[3], Francesc Calafell[1], David Comas[1]*

1. Departament de Medicina i Ciències de la Vida, Institut de Biologia Evolutiva (CSIC-UPF), Universitat Pompeu Fabra, 08003, Barcelona, Spain
2. Facultat de Sociologia. Universitat Autònoma de Barcelona, Barcelona, Spain
3. Facultat de Geografia i Història. Universitat de Barcelona, Barcelona, Spain

FIGURES:

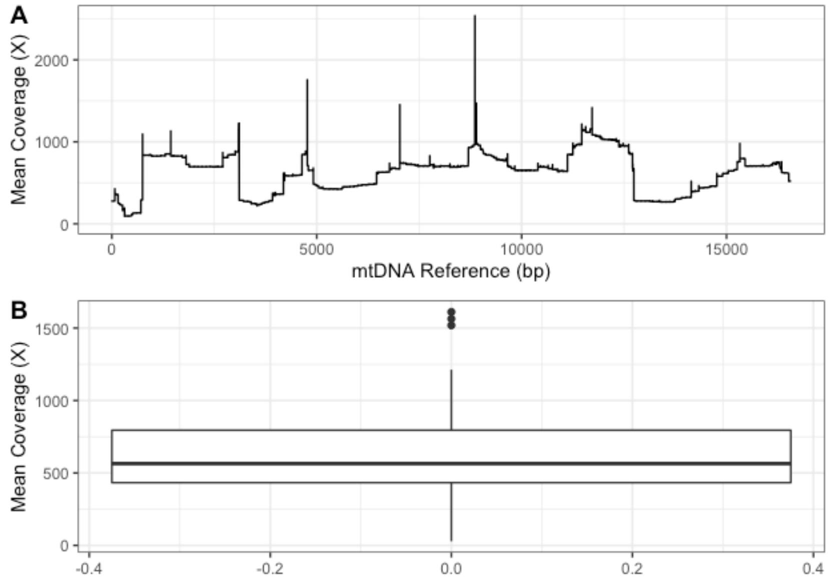

Figure 1 Mean coverage across the mtDNA sequence (A) and mean coverage per individual (B) for the 144 Iberian Roma samples.

Roma Founder Lineages:

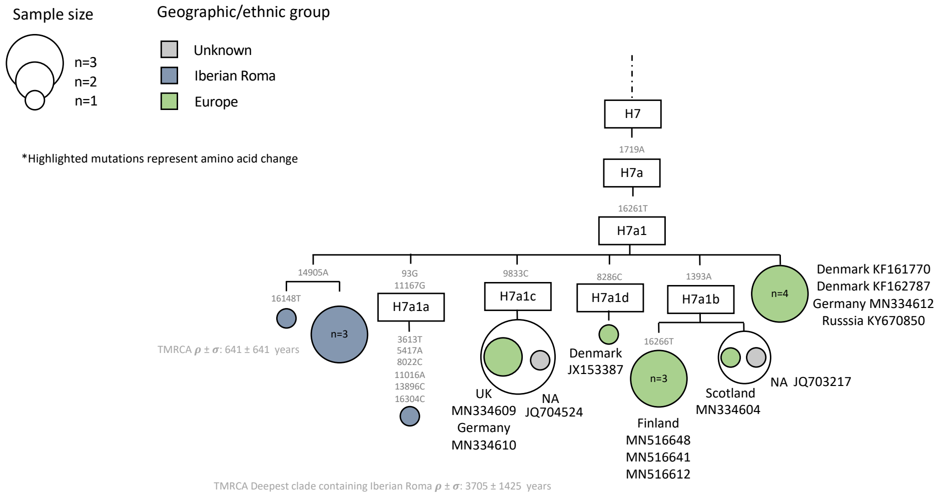

Figure 2 Maximum parsimony tree of the haplogroup H7a1 Roma mitogenomes. The mtDNA variants are indicated along the branches of the phylogenetic tree.

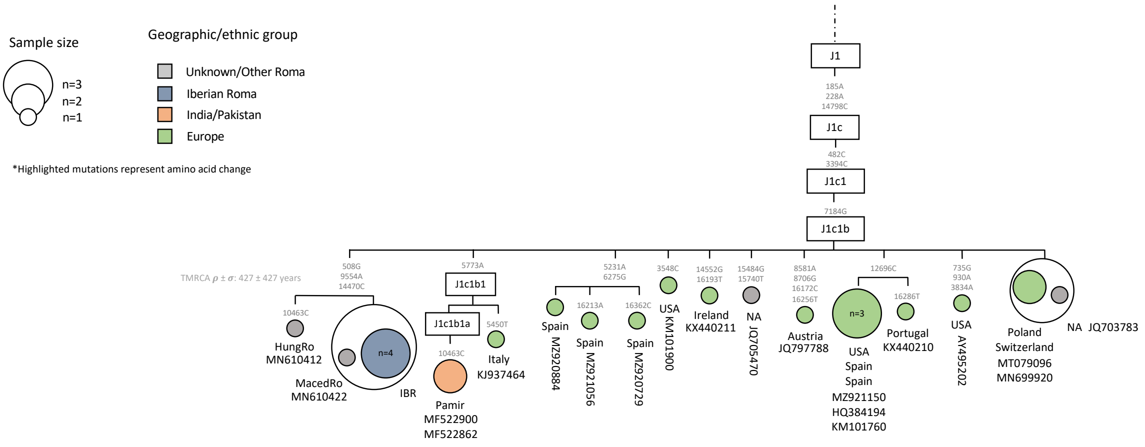

Figure 3 Maximum parsimony tree of the haplogroup J1c1b Roma mitogenomes. The mtDNA variants are indicated along the branches of the phylogenetic tree. The IBR tag refers to Iberian Roma, and ‘HungRo’ and ‘MaceRo’ refer to Hungarian and Macedonian Roma respectively.

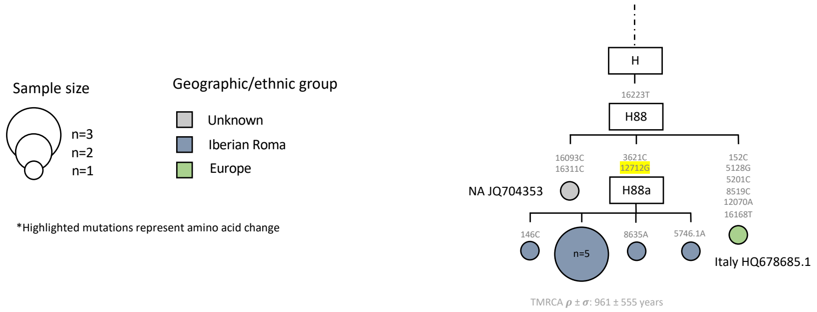

Figure 4 Maximum parsimony tree of the haplogroup H88a Roma mitogenomes. The mtDNA variants are indicated along the branches of the phylogenetic tree. The highlighted mutations represent non-synonymous mutations.

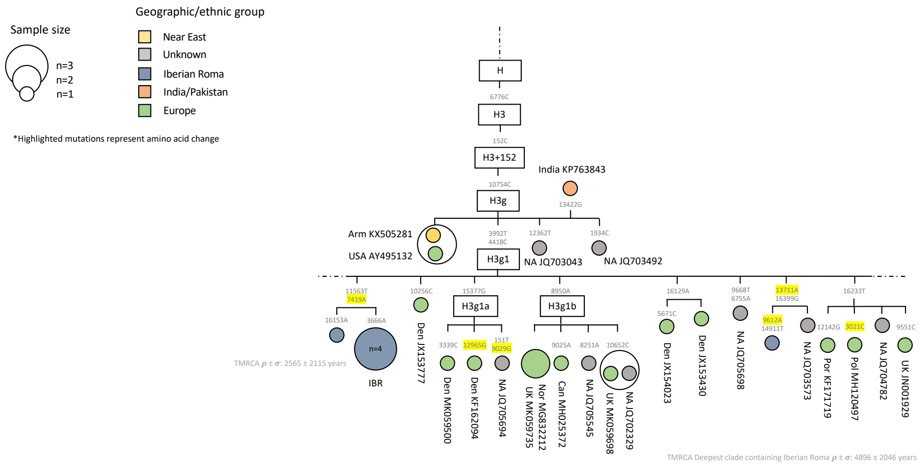

Figure 5 Maximum parsimony tree of the haplogroup H3g1 Roma mitogenomes. The mtDNA variants are indicated along the branches of the phylogenetic tree. The highlighted mutations represent non-synonymous mutations. The IBR tag refers to Iberian Roma.

Maximum Parsimony trees for all the haplogroups reaching the threshold frequency of 0.0278 in the geographic sub-divisions of Iberian non-Roma.

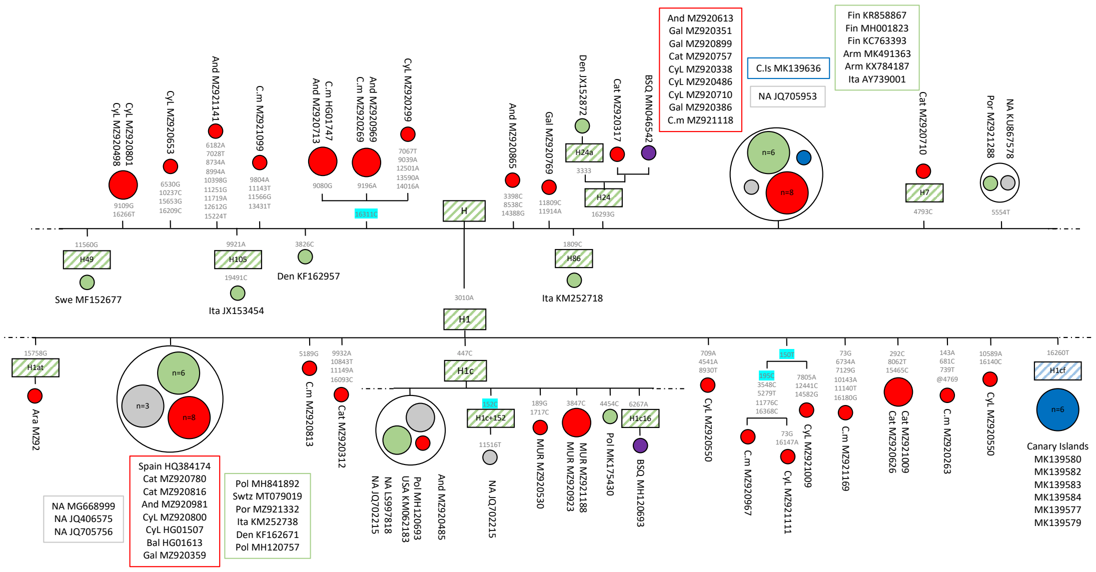

Figure 6 Maximum Parsimony tree for H, H1 and H1c sequences in the Iberian non-Roma dataset and the proximal phylogenetic context. Cat= Catalonia, And=Andalusia, CyL=Castilla and Leon, Bal=Balearic Islands, C.m=Castilla la Mancha, Gal=Galicia, BSQ=Basque Country, Pol=Poland, Den=Denmark, Ita=Italy, Swe=Sweden, Fin=Finland, Arm=Armenia, Por=Portugal. Highlighted mutations represent highly recurrent mutations according to Soares et al 2009 [1].

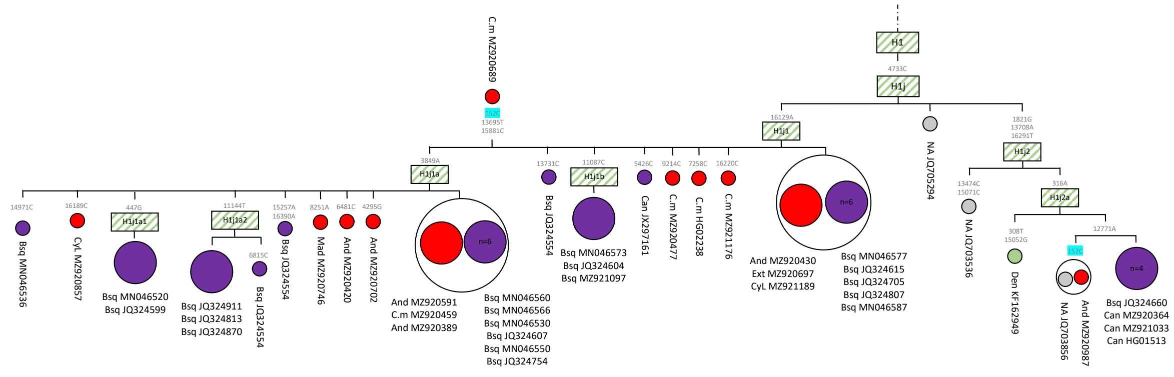

Figure 7 Maximum Parsimony tree for H1j1, H1j2a sequences in the Iberian non-Roma dataset and the proximal phylogenetic context. And=Andalusia, CyL=Castilla and Leon, C.m=Castilla la Mancha, Mad=Madrid, Ext=Extremadura Bsq=Basque Country, Can=Cantabria, Den=Denmark. Highlighted mutations represent highly recurrent mutations according to Soares et al 2009 [1].

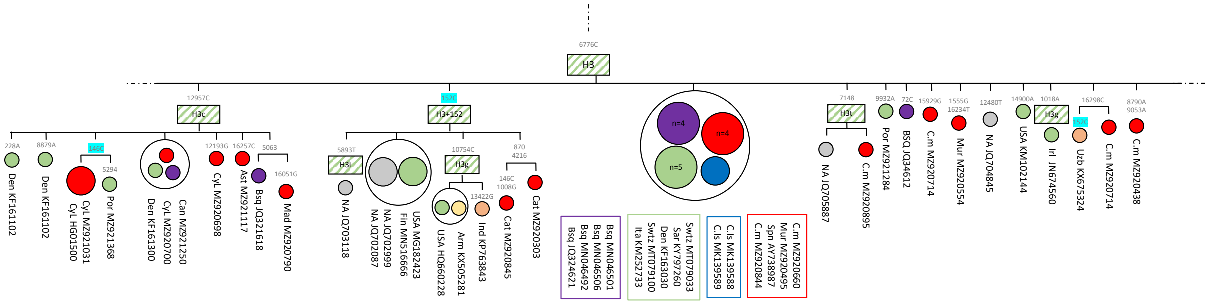

Figure 8 Maximum Parsimony tree for H3, H3+152 and H3c sequences in the Iberian non-Roma dataset and the proximal phylogenetic context. Cat= Catalonia, Mur=Murcia, CyL=Castilla and Leon, Mad=Madrid, C.m=Castilla la Mancha, Ast=Asturias, BSQ=Basque Country, Irl=Ireland, Sar=Sardinian, C.Is=Canary Islands, Switz=Switzerland, Den=Denmark, Ita=Italy, Swe=Sweden, Fin=Finland, Arm=Armenia, Por=Portugal. Highlighted mutations represent highly recurrent mutations according to Soares et al 2009 [1].

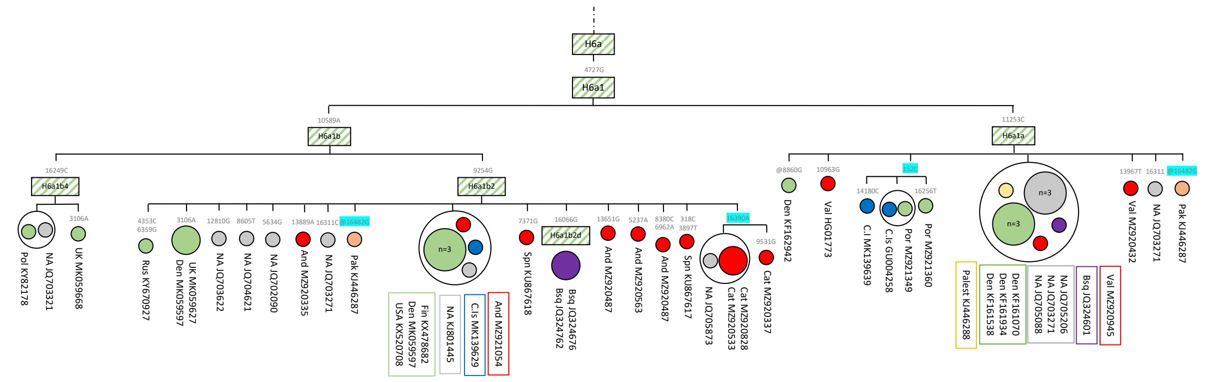

Figure 9 Maximum Parsimony tree for H6a1a, H6a1b sequences in the Iberian non-Roma dataset and the proximal phylogenetic context. Cat= Catalonia, And=Andalusia, Val=Valencia, Bsq=Basque Country, Pol=Poland, Den=Denmark, Pak=Pakistan, Russ=Russia, Fin=Finland, Por=Portugal, Palest=Palestine. Highlighted mutations represent highly recurrent mutations according to Soares et al 2009 [1].

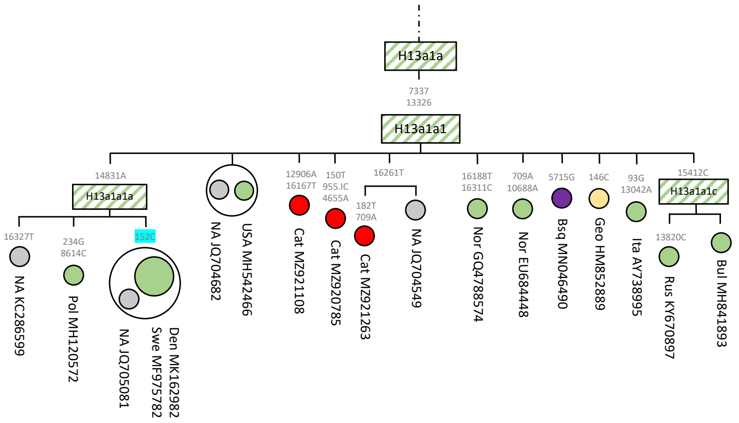

Figure 10 Maximum Parsimony tree for H13a1a1 sequences in the Iberian non-Roma dataset and the proximal phylogenetic context. Cat= Catalonia, Bsq=Basque Country, Pol=Poland, Den=Denmark, Ita=Italy, Swe=Sweden, Nor=Norway, Fin=Finland, Bul=Bulgary,Geo=Georgia, Por=Portugal. Highlighted mutations represent highly recurrent mutations according to Soares et al 2009 [1].

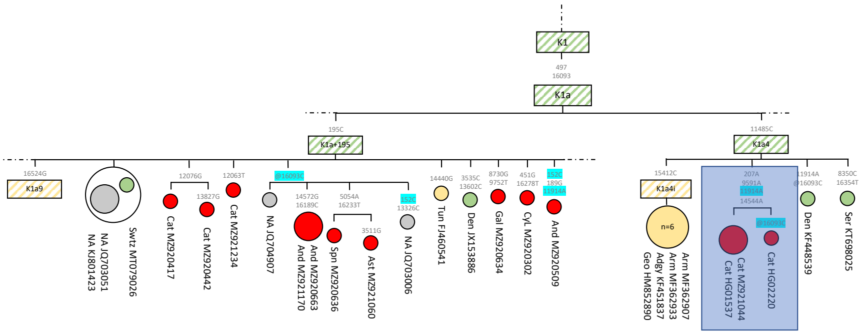

Figure 11 Maximum Parsimony tree for K1a+195 and K1a4 sequences in the Iberian non-Roma dataset and the proximal phylogenetic context. Cat= Catalonia, And=Andalusia, CyL=Castilla and Leon, Gal=Galicia,Ast=Asturias, Den=Denmark, Ser=Serbia, Switz=Switzerland, Arm=Armenia, Geo=Goergia, Adgy=Adygea, Tun=Tunisia. Highlighted mutations represent highly recurrent mutations according to Soares et al 2009 .

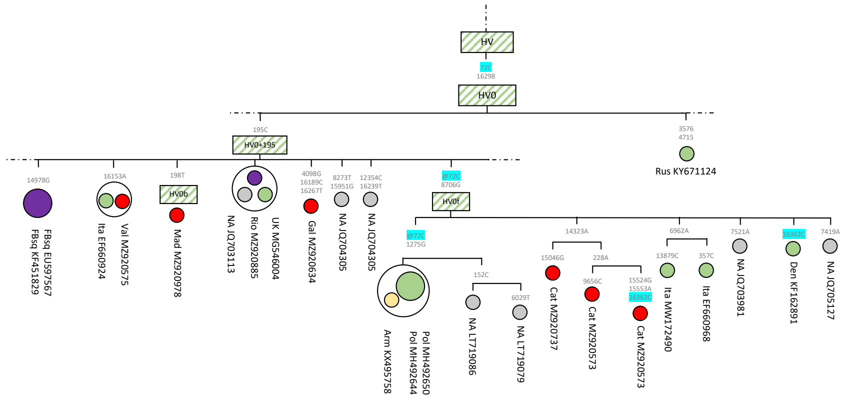

Figure 12 Maximum Parsimony tree for HV0+195 and HV0f sequences in the Iberian non-Roma dataset and the proximal phylogenetic context. Cat= Catalonia, Gal=Galicia,Mad=Madrid,Val=Valencia,Rio=Rioja, Bsq=Basque Country, Pol=Poland, Den=Denmark, Ita=Italy,Rus=Russia, Arm=Armenia. Highlighted mutations represent highly recurrent mutations according to Soares et al 2009 [1].

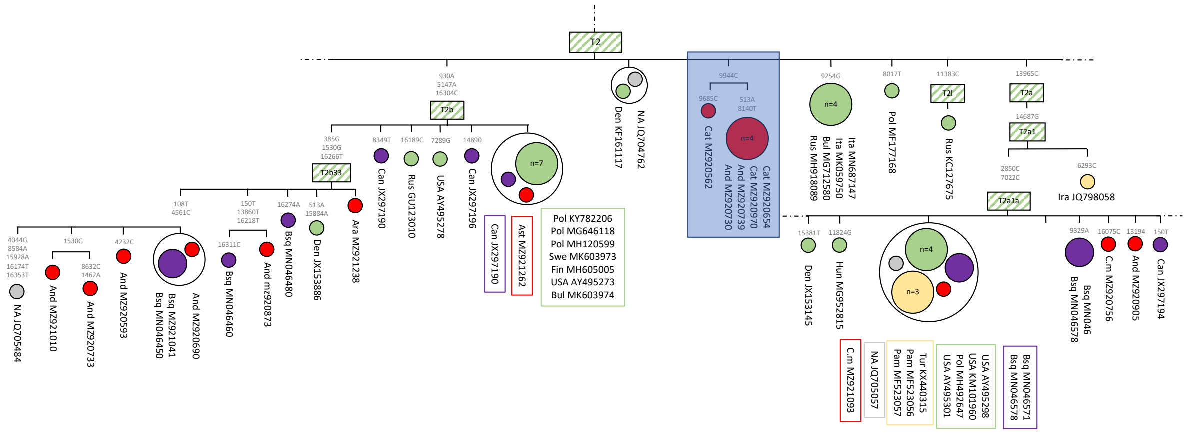

Figure 13 Maximum Parsimony tree for T2, T2a1 and T2b33 sequences in the Iberian non-Roma dataset and the proximal phylogenetic context. Cat= Catalonia, And=Andalusia, Bal=Balearic Islands, C.m=Castilla la Mancha, Ast=Asturias, Bsq=Basque Country, Can=Cantabria, Pol=Poland, Den=Denmark, Ita=Italy, Swe=Sweden, Fin=Finland, Bul=Bulgaria, Hun=Hungary, Tur=Turkey, Pam=Pamir, Ira=Irak, Rus=Russia, Por=Portugal.

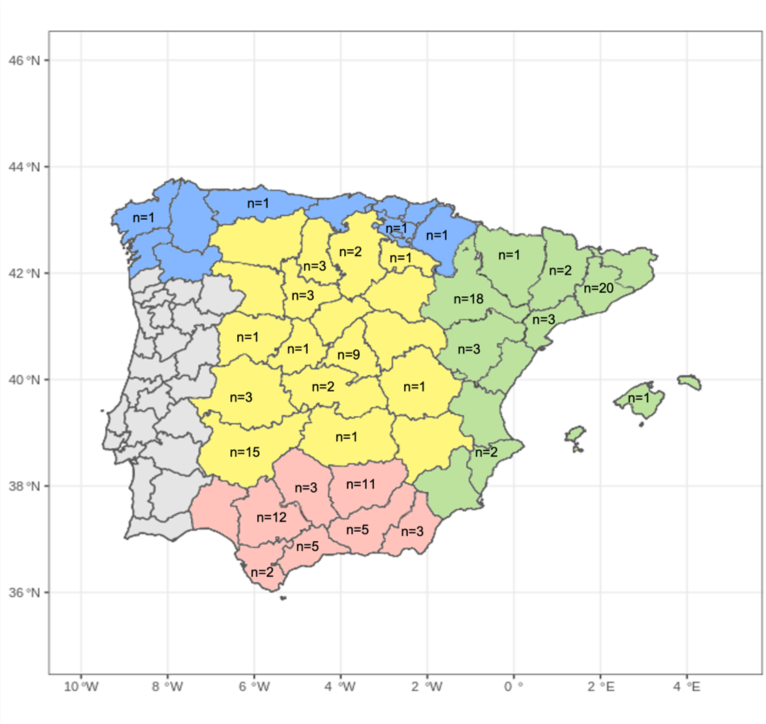

Figure 14 Map of the distribution of the Iberian Roma individuals according to the grandmother's birthplace. Colors represent the grouping done for analyzing substructure of Iberian Roma within the Iberian Peninsula. Map created by using the 'eurostat' package in R [2].

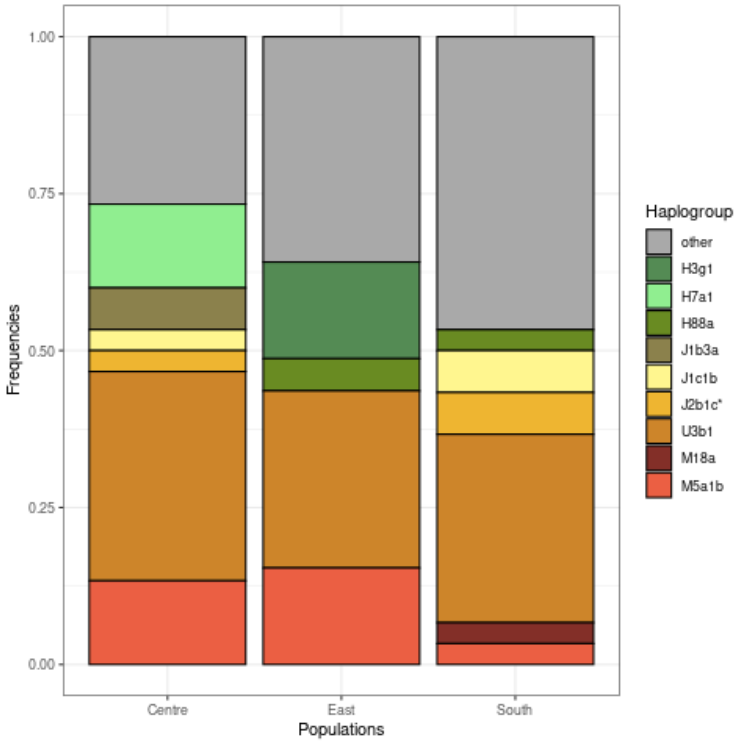

Figure 15 Roma mtDNA Haplogroup composition for each of the main Iberian regions represented in our samples

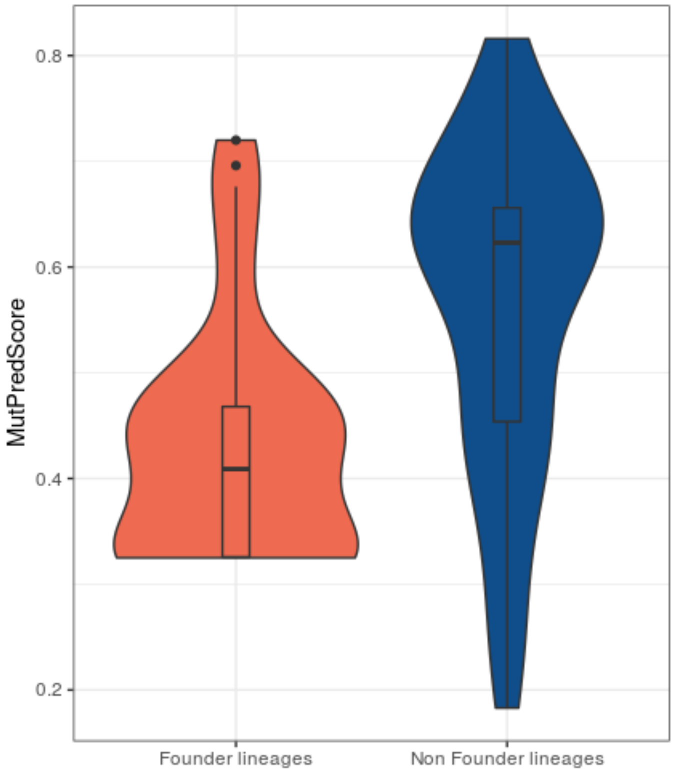

Figure 16 Violin plots of the MutPred pathogenicity score for the non-synonymous mutations in the Iberian Roma founder lineages and Iberian Roma non-founder lineages.

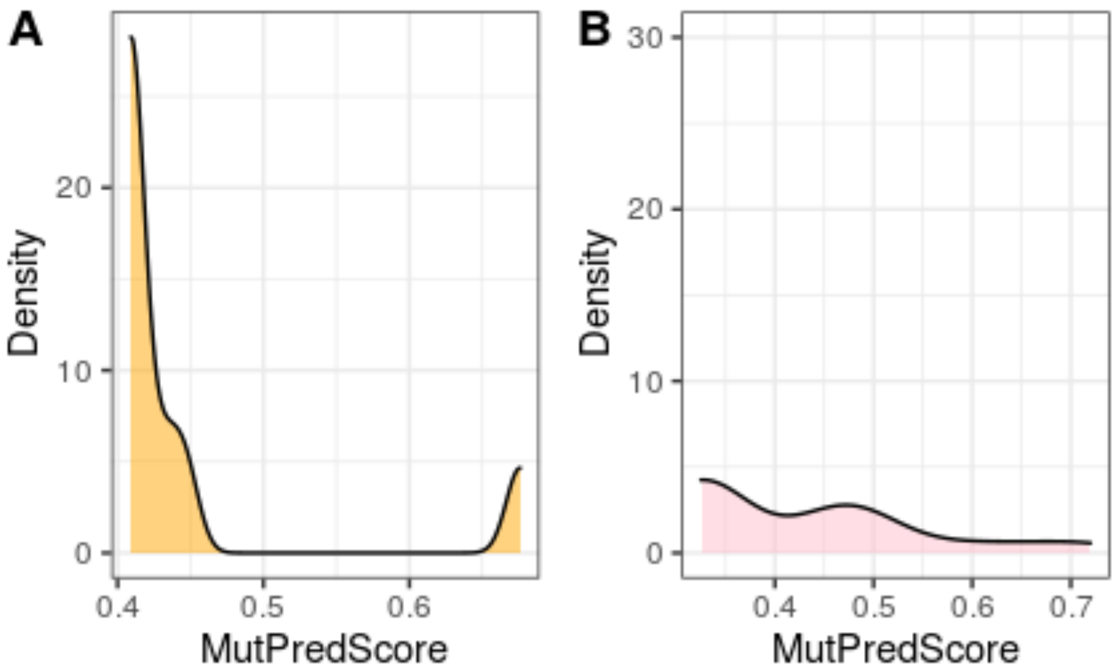

Figure 17 Density plots of the MutPred pathogenicity score for the non-synonymous mutations in the Iberian Roma South Asian founder lineages (A) and Iberian Roma European founder lineages (B).

TABLES

Primer	Primer sequences	Position
L638	5’ TAGACGGGCTCACATCACC 3’	620 - 638
H5043	5’ GGGTTGTACGGTAGAACTGC 3’	5043 - 5063
L4517	5’ CTACTCTACCATCTTTGCAGGC 3’	4495 - 4517
H9001	5’ CAGTAATGTTAGCGGTTAGGCG 3’	9000 - 9022
L8599	5’ GCCGCAGTACTGATCATTC 3’	8581 - 8599
H13187	5’ CTGCTGCGAACAGAGTGG 3’	13169 - 13187
L12852	5’ CCTATACAACCGTATCGGCG 3’	12852 - 12872
H1232	5’ CTGAGCAAGAGGTGGTGAGG 3’	1232 - 1252

Table 1 Primers used for the mtDNA amplification

Reagents (per sample reaction) and Sample	Denaturalization (Temp/Time)	Cyclic denaturalization, annealing and elongation (Temp/ Time)	No. of cycles	Final elongation and final cooling (Temp/ Time)
22.5 µl Platinum PCR Supermix<br>0.5 µl (10µM) Primer F<br>0.5 µl (10µM) Primer R<br>4 µl (5 ng/µl) DNA	94ºC / 2 min	94ºC / 30 sec<br>60ºC / 30 sec<br>68ºC / 5 min	35	68ºC / 10 min<br>4ºC / ∞

Table 2 Conditions for the PCR amplification for every primer pair.

**Populations**	**Number of samples**	**Frequent haplogroups**
Andalusia	227	H, H1j1, H6a1b, K1a+195, T2b33
Catalonia/Balearic Islands	138	H1, H6a1b, K1a+195, H13a1a1, H3+132, HV0f, K1a4, T2
C. Mancha	103	H, H1, H1j1, H3, T2a1
Castilla and Leon	171	H, H1, H3c
Madrid	42	-
Cantabrian Region	63	H1j2a
Aragon	51	-
Murcia	70	H1c, H3
Valencia	59	H6a1a
Galicia	49	H

Table 3 Geographic grouping of the Iberian non-Roma dataset , with the number of samples per group and the haplogroups with a frequency higher than 0.0278 (threshold used to inspect for founder lineages).

	South	East	Centre	North	IBnR	IBR
No. of Samples	227	317	379	143	1066	144
No. of haplotypes	225	305	370	139	1019	72
π (Nucleotide Diversity)	0.00162 ± 0.0008	0.00149 ± 0.0007	0.00137 ± 0.0007	0.00156 ± 0.0008	0.00125 ± 0.0006	0.0017 ± 0.0008
Haplotype Diversity	0.99992 ± 0.0003	0.9998 ± 0.0002	0.99987 ± 0.0001	0.9996 ± 0.0006	0.9999 ± $3.7x10^{-5}$	0.969 ± 0.007

Table 4 Diversity summary statistics for the different Iberian non-Roma geographic groups and the Iberian Roma population.

	**South**	**East**	**Center**	**North**	**Total IBR**
No. of Samples	41	50	42	4	144*(7 samples NA origin)
No. of haplotypes	38	33	37	4	72
π (Nucleotide Diversity)	0.00172 ± 0.0008	0.00136 ± 0.0006	0.00153 ± 0.0007	0.00199 ± 0.001	0.00173 ± 0.0008
Haplotype Diversity	0.996 ± 0.004	0.975 ± 0.009	0.994 ± 0.005	1 ± 0.125	0.969 ± 0.007

Table 5 Diversity indexes of Roma, for the different regions in the Iberian Peninsula.

Lay Summary

Roma, also known with the misnomer of 'Gypsies', are the largest ethnic minority in Europe. They arrived in Europe around the 11th century (CE) but their origin was rather mysterious. Thanks to historic, linguistic and genetic studies, the origin of Roma was traced back to the Punjab region in Northwestern India around 1500 years ago. The proto-Roma left the Punjab and travelled the Middle Eastern territories of Persia. Thanks to genetic studies and linguistics, we know that their dispersion though the Middle East was probably fast, but they stayed in the Caucasus region for a considerable amount of time. They entered Europe through the Balkans and afterwards, persecution and discrimination probably triggered their dispersion across all European countries. The Roma travelled in small sized groups in Europe which caused population bottlenecks, and as a consequence, the different groups started to differentiate, both genetically and culturally.

In the present study, we tried to analyze the maternal genetic diversity of Iberian Roma. We analyzed the maternal genetic diversity by sequencing the mitochondrial DNA (mtDNA) of 144 Roma individuals from different parts of Spain. The analysis of the mtDNA enables to understand the population history of the women ancestors of the present-day populations, since the mtDNA is a molecule just inherited from our mothers.

The results of the study show that Iberian Roma contain a high amount (65%) of Roma specific maternal lineages. Roma acquired these lineages by assimilating women throughout their diaspora. We observe that some of these Roma specific founder lineages have an Indian origin, some other have a Middle Eastern/Caucasian origin, and most of their founder maternal lineages have a European origin. For the sake of comparison, we repeated the analysis with the general Spanish population and discovered that they had just a 0.7% of Spanish specific lineages. The specific geographic origin of most European Roma specific lineages is difficult to know, since the most related lineages can be found all across the continent. However, considering that we observe that Roma from different countries also contain these lineages, it is most likely that Roma obtained most of these maternal lineages early in their arrival in Europe and before the split of the different Roma subgroups.

We also analyzed the genetic substructure within the Iberian Peninsula. We found no significant differences between Central Iberian, Eastern Iberian and Southern Iberian Roma. The nomadic tradition of Roma might have contributed to homogenize the Iberian Roma population. However, genome wide studies were able to detect some substructure within the Iberian Roma.

Finally, we tested whether the Roma mitochondrial founder lineages could be more pathogenic than Roma non-founder lineages. We observe that the non-founder lineages have a higher predicted pathogenicity compared to the founder lineages.

This study helps to understand the impact of founder effects in the Present-day Roma from a maternal perspective and gives us information about the history and genetic diversity of Iberian Roma.

Questions and Answers of the Study:

Why do you conduct population genetic studies?

Population genetic studies help, together with disciplines like history, anthropology or linguistics, to better understand the history of present-day populations. Furthermore, historically ignored and minorized populations might benefit from genetic studies since historical records are not as detailed as for more stablished populations. Besides, it also helps to understand the genetic variability within populations, which gives us information about the prevalence of genetic medical conditions that can affect populations.

Why do you use mitochondrial DNA?

First, the number of mtDNA copies per cell is remarkably higher in comparison to the nuclear DNA. Second it has a higher mutation rate, which enables the accumulation of more genetic variability, which is the raw material for population genetic inferences. Third, it has a small size (around 16500 base pairs) and it is easy to sequence. And fourth, it is maternally inherited as a unit. This makes it a useful tool to study sex specific genetic patterns.

What are the main findings of the study?

We found that Iberian Roma contain a substantially large amount of Roma founder lineages in contrast with the almost absence found in the non-Roma Iberians. Roma founder lineages are Roma specific lineages that they adopted during their diaspora and which frequencies grew as a consequence of randomness after a genetic bottleneck (genetic drift). Within the Roma founder lineages, we found and detailed lineages with Northwestern Indian origin, which reflects their origin in this region; Middle Eastern lineages adopted by Roma throughout their diaspora; lineages from the Balkan region, which reflects their stay in the region shortly after their arrival in Europe; and finally, unspecific European lineages also adopted by Roma along their travel within Europe.

What are the limitations of the study?

First, it is necessary to bear in mind that the mitochondrial DNA represents just a single ancestral genetic genealogy (the matrilineal genealogy), therefore some inferences such as the population structure analysis are limited by this making the use of genome wide markers more trustful.

The definition of "founder lineage" we used to determine whether a group of linages is, indeed, a founder group, can be interpreted as arbitrary. To overcome this limitation, we repeated the analysis on the Iberian non-Roma population (with an even more restrictive definition of founder lineage, since we divided the Iberian samples into geographical sub-groups), which gives us a way to contextualize the results.

Besides, the inference on the geographical origin of the lineages is very dependent on the samples available on the scientific literature. However, we tried to not be jumping to conclusions about the origin of founder lineages in cases where the phylogeographic context was not clear. Finally, when assessing the pathogenicity of the lineages, it we just considered the mtDNA coding region and ignored tRNA mutations or variant in the control region. Thus, we might not be capturing the complete picture of the pathogenicity of mitochondrial lineages.

REFERENCES:

[1] P. Soares *et al.*, “Correcting for Purifying Selection: An Improved Human Mitochondrial Molecular Clock,” *Am J Hum Genet*, vol. 84, no. 6, p. 740, Jun. 2009, doi: 10.1016/J.AJHG.2009.05.001.

[2] L. Lahti, J. Huovari, M. Kainu, and P. Biecek, “Retrieval and analysis of eurostat open data with the eurostat package,” *R Journal*, vol. 9, no. 1, pp. 385–392, Jun. 2017, doi: 10.32614/RJ-2017-019.
